# Prevalence of Common Gynecological Conditions in the Middle East: Systematic Review and Meta-Analysis

**DOI:** 10.3389/frph.2021.661360

**Published:** 2021-04-06

**Authors:** Mira Mousa, Moamar Al-Jefout, Habiba Alsafar, Shona Kirtley, Cecilia M. Lindgren, Stacey A. Missmer, Christian M. Becker, Krina T. Zondervan, Nilufer Rahmioglu

**Affiliations:** ^1^Nuffield Department of Women's and Reproductive Health, Endometriosis CaRe Centre, University of Oxford, Oxford, United Kingdom; ^2^Department of Obstetrics and Gynaecology, College of Medical and Health Sciences, United Arab Emirates University, Al Ain, United Arab Emirates; ^3^Department of Obstetrics and Gynaecology No. 1. Moscow, I.M. Sechenov First Moscow State Medical University, Moscow, Russia; ^4^Center for Biotechnology, Khalifa University of Science and Technology, Abu Dhabi, United Arab Emirates; ^5^Department of Genetics and Molecular Biology, College of Medicine and Health Sciences, Khalifa University of Science and Technology, Abu Dhabi, United Arab Emirates; ^6^Nuffield Department of Orthopaedics, Rheumatology, and Musculoskeletal Sciences, Centre for Statistics in Medicine, University of Oxford, Oxford, United Kingdom; ^7^Li Ka Shing Centre for Health Information and Discovery, The Big Data Institute, University of Oxford, Oxford, United Kingdom; ^8^Nuffield Department of Medicine (C.M.L.), Wellcome Centre for Human Genetics, University of Oxford, Oxford, United Kingdom; ^9^Department of Epidemiology, Harvard T.H. Chan School of Public Health, Boston, MA, United States; ^10^Department of Obstetrics, Gynaecology, and Reproductive Biology, College of Human Medicine, Michigan State University, Grand Rapids, MI, United States; ^11^Wellcome Centre for Human Genetics, University of Oxford, Oxford, United Kingdom

**Keywords:** gynecological disease, epidemiology, global health, Middle East, polycystic ovary syndrome, endometriosis, uterine fibroids, adenomyosis

## Abstract

**Introduction:** High prevalence of gynecological conditions in women of Middle Eastern origin is reported, likely due to regional risk factors and mediators. The objective of this systematic review and meta-analysis is to investigate the prevalence of polycystic ovary syndrome (PCOS), endometriosis, uterine fibroids, and adenomyosis in women of Middle Eastern origin.

**Methods:** MEDLINE, EMBASE, PsycINFO, Global Health, and Google Scholar databases were searched from database inception until 14 February 2021 to identify relevant studies. Peer-reviewed research articles that reported the prevalence of PCOS, endometriosis, uterine fibroids, and adenomyosis in the Middle Eastern population were written in English or Arabic. The primary outcome was the estimated pooled prevalence of PCOS, endometriosis, uterine fibroids, and adenomyosis in the Middle Eastern populations. The secondary outcome was to assess the evidence in the data for the presence of heterogeneity, by conducting subtype-pooled analysis of prevalence estimates of the conditions. Total weighted prevalence was calculated via Freeman–Tukey arcsine transformation and heterogeneity through the *I*^2^ statistic. Quality control was performed using GRADE criteria.

**Results:** A total of 47 studies, 26 on PCOS, 12 on endometriosis, eight on uterine fibroids, and seven on adenomyosis, were included. The pooled prevalence of PCOS diagnosed according to the NIH criteria was 8.9% (95% CI: 6.5–11.7; prevalence range: 4.0–27.6%), with a higher prevalence from the Gulf Arab states (18.8%, 95% CI: 9.5–30.3; range: 12.1–27.6%). According to the Rotterdam criteria, the pooled prevalence of PCOS was 11.9% (95% CI: 7.1–17.7; range: 3.4–19.9%) with studies limited to the Persian and Levant regions. Endometriosis was diagnosed in 12.9% (95% CI: 4.2–25.4; range: 4.2–21.0%) of women undergoing laparoscopy, for any indication. Uterine fibroid and adenomyosis prevalence of women was 30.6% (95% CI: 24.9–36.7; range: 18.5–42.6%) and 30.8% (95% CI: 27.1–34.6, range: 25.6–37.7%), respectively. Heterogeneity was present between studies due to statistical and methodological inconsistencies between studies, and quality of evidence was low due to sample size and unrepresentative participant selection.

**Conclusion:** This is the first review that has reported the prevalence of gynecological diseases in the Middle Eastern population, suggesting that gynecological morbidity is a public health concern. Due to the health disparities in women, further research is required to understand the relative roles of environmental and genetic factors in the region to serve as a benchmark for evaluation and comparative purposes with other populations.

## Introduction

Polycystic ovary syndrome (PCOS), endometriosis, uterine fibroids, and adenomyosis are common benign gynecological conditions that affect women of reproductive age. They are often associated with dysfunctional uterine bleeding, pelvic pain, subfertility, psychological morbidity, and comorbid diseases (1–4). Genetic and environmental factors contribute to the risk of gynecological conditions, but none are currently specific enough to be clinically relevant. In addition, little is known regarding the reasons for the heterogeneity in symptomatology and contributing factors attributable to gynecological conditions in different populations of women, especially women of Middle Eastern origin. Table 1 summarizes the clinical epidemiology of PCOS, uterine fibroids, endometriosis, and adenomyosis, based on global statistics of mainly European ancestry populations.

**Table 1:** Prevalence estimates, symptoms, diagnostic criteria, treatment method, risk factors, and average annual cost estimate of PCOS, endometriosis, adenomyosis, and uterine fibroids.

**Condition**	**Prevalence**	**Symptoms**	**Diagnosis**	**Treatment**	**Risk factors**	**Annual patient cost estimate in European populations**
Polycystic Ovary Syndrome	- NIH criteria (~5–9%)^a, b^ - Rotterdam Criteria (~10–20%)^b, c^	- Hyperandrogenism^d, e, f^ - Menstrual irregularity^d, e, f^ - Oligo-anovulation^d, e, f^ - Hirsutism^d, e, f^ - Obesity^d, e, f^ - Dyslipidemia^d, e, f^ - Infertility^d, e, f^	- Ultrasonography^d, e, f^ - Biochemical^d, e, f^ - Clinical evidence (NIH criteria and Rotterdam criteria)^d, e, f^	- Medical treatment (suppressive hormonal therapy)^d, e, f^ - Ovarian drilling^d, e, f^	- Early age of menarche^d, f, g^ - Ovulatory dysfunction^d, f, g^ - High body mass index^d, f, g^ - Insulin resistance^d, f, g^ - Metabolic syndrome features^d, f, g^ - Altered glucose homeostasis ^d, f, g^ - Family history ^d, f, g^	€4,413 to €7,293^h, i^
Endometriosis	5–10%^j^	- Dysmenorrhea^j, k, l, m, n, o^ - Dyspareunia^j, k, l, m, n, o^ - Dyschezia^j, k, l, m, n, o^ - Dysuria^j, k, l, m, n, o^ - Infertility^j, k, l, m, n, o^ - Abnormal uterine bleeding^j, k, l, m, n, o^ - Chronic pelvic pain^j, k, l, m, n, o^	- Laparoscopy or laparotomy^j, n^ - Magnetic resonance imaging ^j, n^ - Ultrasonography^j, n^	- Surgical removal of lesions^j, n^ - Medical treatment (suppressive hormonal therapy)^j, n^	- Early age of menarche^n, p, q^ - Short menstrual cycles^n, p, q^ - Increased menstrual flow^n, p, q^ - Low body mass index^n, p, q^ - Family history^n, p, q^ - Late menopause^n, p, q^	€9,579^r^
Adenomyosis	14–57%^s^	- Menorrhagia^t, u^ - Dysmenorrhea^t, u^ - Endometrial polyps^t, u^ - Leiomyomata uteri^t, u^ - Chronic pelvic pain^t, u^	- Transvaginal ultrasonography^t, u^ - Magnetic resonance imaging^t, u^ - Hysterectomy^t, u^	- Surgical extirpation^u^ - Medical treatment (Suppressive hormonal therapy)^u^	- Increase in age^t^ - Number of births^t^ - Smoking^t^ - Previous uterine surgery^t^	€6,719.9^w^
Uterine fibroids	5–30%^x^	- Abnormal uterine bleeding^x, y^ - Pelvic pain^x, y^ - Pelvic mass^x, y^ - Dyspareunia^x, y^ - Infertility^x, y^	- Magnetic resonance imaging ^x, y^ - Ultrasonography^x, y^	- Medical treatment (suppressive hormonal therapy)^x, y^ - Uterine artery embolization^x, y^	- Ethnicity ^x, y^ - Early age of menarche ^x, y^ - High body mass index ^x, y^ - Parity^x, y^ - Smoking^x, y^	€12,654 to €30,075^z^

*(5)^a^; (6)^b^; (7)^c^; (2)^d^; (8)^e^; (9)^f^; (10)^g^; (11)^h^; (12)^i^; (4)^j^; (13)^k^; (14)^l^; (15)^m^; (16)^n^; (17)°; (18)^p^; (19)^q^; (20)^r^; (21)^s^; (22)^t^; (23)^u^; (24)^v^; (25)^w^; (3)^x^; (26)^y^; (27)^z^*.

The Middle East represents 6.8% of the world's population (23 countries), yet it contributes <1% toward scientific research (28–31). Cross-population comparisons and regional differences, influenced by geographic, cultural, socioeconomic, genetic, and environmental factors, may alter health outcome measures associated with prevalence, symptomatology, diagnosis, and management of gynecological phenotypes. A high prevalence of gynecological conditions has been suggested in women of Middle Eastern origin, possibly due to consanguinity, obesity rates, environmental toxins from war exposure, and lack of awareness of reproductive health among adolescence (32–37).

The Middle East is a transcontinental region that consists of the Gulf Cooperative Council (GCC) Region (Bahrain, Kuwait, Oman, Qatar, Saudi Arabia, United Arab Emirates, Yemen), the Levant region (Cyprus, Iraq, Israel, Jordan, Lebanon, Palestine, Syria, Turkey), the North African region (Algeria, Egypt, Libya, Morocco, Somalia, Sudan, Tunisia), and the Persian region (Iran). The Middle Eastern populations have different degrees of genetic admixture, and higher prevalence of globally rare genetic variations with a highly conserved gene pool, due to consanguineous marriages and intercontinental migration (38–41). Consanguineous marriages account for 20–50% of marriages in the Arab ancestry Middle Eastern populations, resulting in higher frequency of recessive Mendelian diseases, large prevalence of deleterious genetic missense variants, and community-specific founder mutations (33, 42, 43). Nevertheless, the Middle East has a very diverse ancestral background, due to intercontinental migration between Africa, Asia, and Europe. Nine Middle Eastern countries have ranked highest in the global obesity statistics, with 70–75% of their populations being obese or overweight, with a 2-fold risk among women vs. men (36, 44–48). Physical inactivity and obesity rates in the region have been associated with gynecological diseases and menstrual disorders (49, 50). Additionally, exposure to war and environmental toxins may alter circulating hormone levels and the immune system of women (51).

This systematic review critically assesses the available evidence and quality of epidemiological studies that focus on the prevalence of common benign gynecological diseases in Middle Eastern populations, to inform public health policy and encourage studies into risk factors and prevention. The primary aim was to conduct a systematic review and meta-analysis of the prevalence of polycystic ovary syndrome, endometriosis, uterine fibroids, and adenomyosis in women of Middle Eastern origin. The secondary aim was to assess the evidence in the data for the presence of heterogeneity by conducting subtype-pooled analysis of prevalence estimates of the conditions.

## Methods

### Search Strategy

This systematic review was reported in accordance with PRISMA (Preferred Reporting Items for Systematic Reviews and Meta-Analyses) guidelines (52, 53) and registered for inclusion in PROSPERO: CRD42019119804.

The electronic bibliographic databases, MEDLINE, EMBASE, PsycINFO, and GLOBAL HEALTH (through the OVID platform), and Google Scholar database, were searched from database inception until 14 February 2021. Search terms included a combination of free-text or controlled vocabulary terms (MeSH and EMTREE) for gynecological conditions including “Polycystic Ovary Syndrome,” “Endometriosis,” “Uterine Fibroids,” and “Adenomyosis.” We combined these with terms related to “Epidemiology” and “Prevalence” -related terms and terms for the “Middle East” -related countries. No other limits were applied to the search to obtain a comprehensive literature including the maximum possible number of studies. Details of the full search strategy used for the MEDLINE database are provided in Supplementary Text 1.

### Eligibility Criteria and Selection of Studies

Peer-reviewed research articles that reported the prevalence of PCOS, endometriosis, uterine fibroids, and adenomyosis in women of Middle Eastern origin were included, in English and Arabic languages. The primary aim was to estimate the pooled prevalence estimate of PCOS, endometriosis, uterine fibroids, and adenomyosis in the Middle East, based on the following diagnostic criteria: NIH (54) and Rotterdam criteria (55) for PCOS; laparoscopic diagnosis for endometriosis; ultrasound sonography, MRI, and/or hysterectomy for adenomyosis; and ultrasound sonography, MRI, and/or hysterectomy for uterine fibroids. The secondary aim was to assess the evidence in the prevalence data for the presence of heterogeneity, in association to the following characteristics: ascertainment criteria (hospital-based/population-based), diagnostic criteria, age group of participants (adolescent/adult), country of origin (GCC region/Levant region/North Africa region/Persian region), type of study design (cross-sectional/cohort study), and availability of health insurance coverage.

The study outcomes to assess the pooled prevalence of PCOS were population-based study with the NIH criteria; population-based study with the Rotterdam criteria; hospital-based study with the Rotterdam criteria for any gynecological indication; and hospital-based study with the Rotterdam criteria for infertility investigation. For the pooled prevalence of endometriosis, distinction was made between population-based studies with a laparoscopic confirmation; hospital-based studies with a laparoscopic confirmation for any gynecological indication; and hospital-based studies with a laparoscopic confirmation for infertility investigation. The pooled prevalence of uterine fibroids and adenomyosis was divided into hospital-based studies with a hysterectomy or imaging diagnosis for any gynecological indication and hospital-based studies with a hysterectomy or imaging diagnosis for abnormal uterine bleeding investigation.

To reduce the risk of selection bias, methodological bias, and heterogeneity, studies were included in the meta-analysis when (i) the sample size involved >100 participants; (ii) diagnostic criteria were described; and (iii) prevalence estimates were reported. Reviews, editorials, and organizational guidelines were excluded to avoid bias toward more frequently cited publications. Case reports/series were excluded given that they use no control group to compare outcomes and have little statistical validity. Conference abstracts were excluded as there was no access to the full report and the studies were not peer-reviewed. To avoid over-representation of cases, when several studies on the same series of participants were published, only the report with the largest sample size was included in the meta-analysis.

### Data Extraction

Retrieved articles were uploaded to EndNote and duplicates deleted. One reviewer (M.M.) reviewed the full library for relevance. A second reviewer (N.R.) reviewed the retrieved articles; inter-reviewer concordance was checked, and the third reviewer (K.Z.) adjudicated discordant results. A second round of review was conducted, and full texts were assessed for inclusion. Data was extracted into a standard form including study design, participant characteristics, ascertainment method, nationality, diagnostic criteria, confounding factors, prevalence estimates, and outcome measures. Missing data were collected where possible by emailing the corresponding author, or through statistical calculations reported in the Cochrane handbook for systematic reviews (56). All procedures conformed to the guidelines for systematic review and meta-analysis of observational studies in epidemiology: the Meta-analysis Of Observational Studies in Epidemiology Checklist (57).

### Assessment of Risk of Bias

The quality control, assurance, and bias of the studies were assessed by two independent reviewers (M.M. and N.R.) using the Grading of Recommendations Assessment, Development and Evaluation (GRADE) criteria (58, 59). The quality of evidence was monitored for each study based on risk of bias, imprecision, inconsistency, indirectness, and publication bias and scored on four levels of evidence: very low, low, moderate, and high. The studies and outcomes were rated down for clinical heterogeneity, methodological heterogeneity, unrepresentative control groups, insufficient diagnostic criteria for case definition, lack of adjustment to confounding variables, unclear selection criteria, sample size, confidence interval around effect estimate, and selection bias.

### Data Synthesis

Statistical analyses were conducted using the metaprop function in R (R package: meta, Schwarzer 2008, Version 2.7.1). Meta-analysis was used to synthesize the pooling proportion estimates of the gynecological condition. The Freeman–Tukey double arcsine transformation was applied for normalizing and variance stabilizing of the proportion sampling distribution. This transformation also provided confidence limits of proportions between zero and one. Heterogeneity among studies was assessed via *I*^2^ statistic. *I*^2^ of 50% or above was designated significant heterogeneity and 30–49% moderate heterogeneity (60). The random-effect model of DerSimonian and Laird was used to estimate the pooled prevalence and its 95% CI, and *I*^2^ heterogeneity was calculated for sensitivity analysis. Significant heterogeneity was explored by performing *post-hoc* subgroup meta-analyses to allow reliable conclusions to be drawn from analyses that involved subgroups. Forest plots demonstrate the pooled prevalence estimates of the meta-analysis results. *P* < 0.05 was used as the threshold for statistical significance.

## Results

### Study Selection and Characteristics

**Figure 1:**
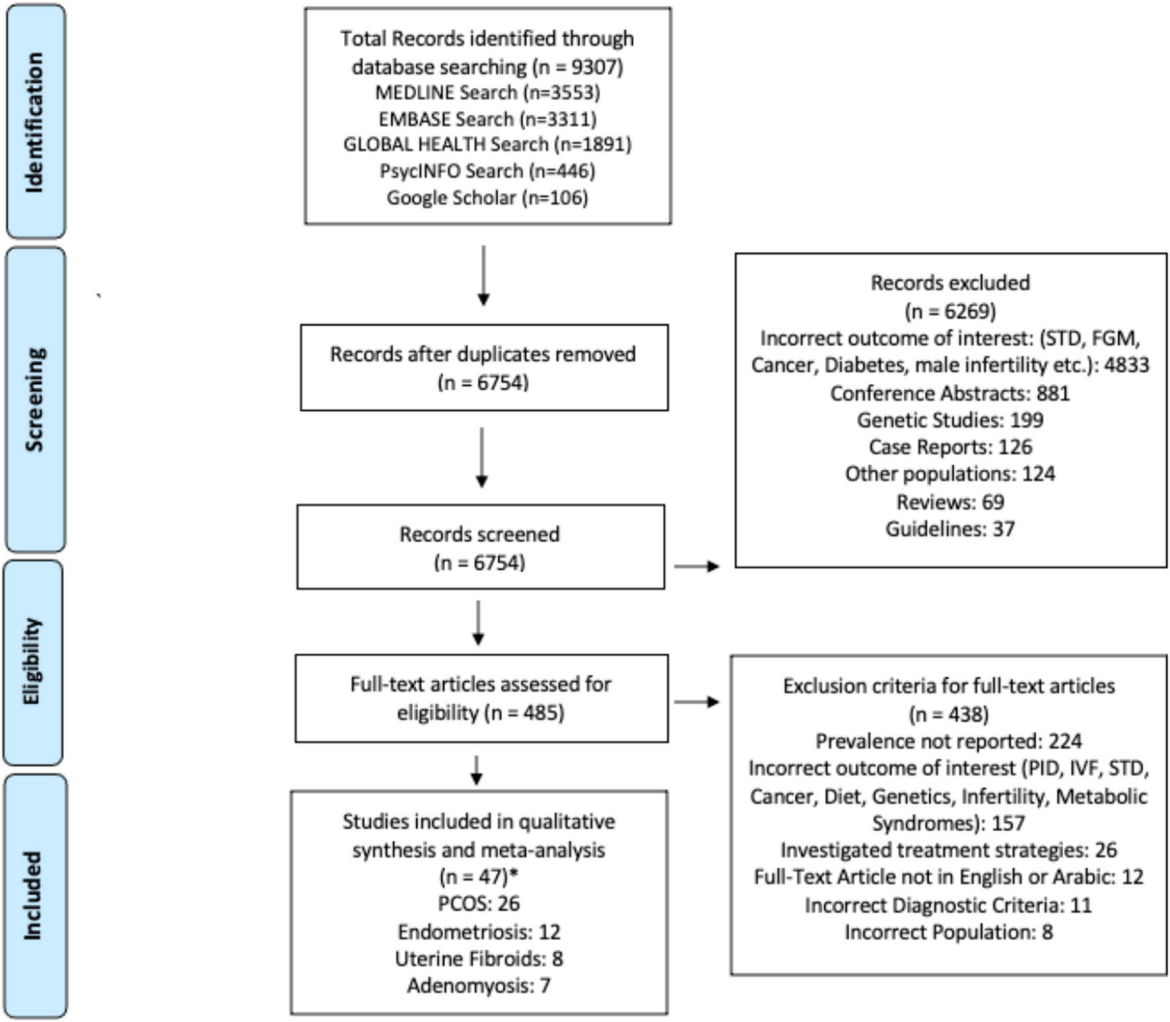
PRISMA Figure. *Six studies reported prevalence estimates for more than one condition.

The search strategy across all databases retrieved 9,307 studies, 6,754 studies after duplicates were removed (Figure 1); 6,269 failed to meet the inclusion criteria based on abstract review. Following a full-text review of 485 studies, 438 were excluded because they did not meet the inclusion criteria. Of the 47 articles, 26 covered prevalence of PCOS, 12 endometriosis, 8 uterine fibroids, and 7 adenomyosis. There were 25 retrospective studies using electronic medical records and 22 cross-sectional studies; 29 were hospital-based and 18 population-based. There were 14 studies published from the GCC region, 14 studies from the Persian region, 14 studies from the Levant region, and 5 studies from the North African region. Detailed characteristics of the studies included are given in Table 2.

**Table 2:** Characteristics of the 47 studies included in the meta-analysis.

**References**	**Country**	**Study period**	**Study design**	**Participant ascertainment**	**Sample size**	**Age Mean ± Sd (Range)**	**Gynecological condition**	**Diagnostic method/criteria**
(61)	Egypt	1998–2005	Retrospective	Electronic records screened for women attending the endoscopy unit at the obstetrics and gynecology department in a University hospital	2,493	26.8 ± 5.3 (Not reported)	Endometriosis	Laparoscopy
(62)	Egypt	2012–2014	Cross-sectional	Adolescent girls with severe dysmenorrhea	220	15.2 ± 3.53 (Not reported)	Endometriosis	Laparoscopy
(63)	Egypt	2011–2012	Cross-sectional	Infertile women visiting a specialized infertility clinic	3,900	27.9 ± 4.9 (Not reported)	PCOS	Rotterdam criteria
(64)	Egypt	2010–2011	Cross-sectional	Infertile women visiting an outpatient clinic in a University hospital	830	26.8 ± 5.4 (18.0–37.0)	PCOS	Rotterdam criteria
(65)	Egypt	2001–2010	Retrospective	Electronic records screened for the investigation of infertility in a University hospital	4,103	27.6 ± 4.8 (Not reported)	Endometriosis; PCOS	Laparoscopy; Rotterdam criteria
(66)	Iran	2012–2013	Cross-sectional study	Women referred for a laparoscopy due to unexplained infertility	383	30.9 ± 5.3 (Not reported)	Endometriosis	Laparoscopy
(67)	Iran	2001–2003	Retrospective	Electronic records screened for women presenting with hirsutism to the dermatology clinic in a University hospital	790	25.9 ± 5.7 (10.0–45.0)	PCOS	Rotterdam criteria
(68)	Iran	2011–2013	Retrospective	Electronic records screened for women who underwent laparoscopy for the investigation of infertility in a hospital	1,282	32.4 ± 4.9 (16.0–46.0)	Endometriosis	Laparoscopy
(69)	Iran	2009	Cross-sectional study	Female high school students	1,850	17.2 ± 0.70 (17.0–18.0)	PCOS	NIH Criteria
(70)	Iran	Not reported	Cross-sectional study	Female high school students	1,549	17.3 ± 0.9 (16.0–20.0)	PCOS	Rotterdam criteria
(71)	Iran	Not reported	Cross-sectional study	Female high school students	1,000	16.0 ± 1.9 (16.0–20.0)	PCOS	NIH criteria
(72)	Iran	2001–2011	Retrospective	Women attending a hospital for a hysterectomy	191	51.6 ± 12.5 (23.0–85.0)	Adenomyosis	Hysterectomy
(73)	Iran	2007–2008	Retrospective	Women attending a hospital for a hysterectomy	100	46.8 ± 7.8 (21.0–75.0)	Adenomyosis; uterine fibroids	Hysterectomy
(74)	Iran	2009	Cross-sectional study	Mandatory pre-marriage screening clinic	820	29.1 ± 7.6 (Not reported)	PCOS	Rotterdam/NIH criteria
(75)	Iran	Not reported	Cross-sectional study	Multi-stage random sampling of random household lists of premenopausal women available in the health department	602	33.2 ± 3.6 (18.0–45.0)	PCOS	Rotterdam/NIH criteria
(76)	Iran	2005–2006	Cross-sectional study	Multistage random sampling of high school students in Tehran	1,430	15.8 ± 1.1 (15.0–18.0)	PCOS	Rotterdam criteria
(77)	Iran	Not reported	Cross-Sectional Study	Random sampling method of participants selected from the Tehran lipid and glucose study	1,002	29.2 ± 8.7 (18.0–45.0)	PCOS	NIH criteria
(78)	Iran	Not reported	Cross-sectional study	Random household lists of premenopausal women available in the health department	929	34.4 ± 7.6 (18.0–45.0)	PCOS	Rotterdam/NIH criteria
(79)	Iraq	2020	Cross-sectional study	Cluster sampling method from young adolescent girls at high schools	900	15.96 ± 0.91 (14.0–18.0)	PCOS	NIH criteria
(80)	Iraq	2017	Cross-sectional study	Women attending an infertility center in a hospital	100	Not reported (21.0–44.0)	PCOS	Rotterdam criteria
(81)	Iraq	2007–2008	Retrospective	Women attending a hospital for a hysterectomy	391	46.6 ± 4.2 (40.0–49.0)	Adenomyosis; uterine fibroids	Hysterectomy
(82)	Israel	1998–2015	Retrospective	Performed from the Maccabi healthcare service database	570,781	40.4 ± 8.0 (15.0–55.0)	Endometriosis	Laparoscopy
(83)	Israel	Not reported	Cross-sectional study	Post-menopausal women attending an out-patient clinic	104	56.3 ± 5.5 (47.0–64.0)	PCOS	Rotterdam criteria
(84)	Jordan	2015	Cross-sectional study	Self-administered questionnaire from random women selected at universities, schools, and shopping centers	1,772	27.9 ± 5.7 (15.0–45.0)	Endometriosis	Laparoscopy
(85)	Jordan	2008–2009	Retrospective	Women attending hospital for hysterectomy	148	46.6 ± 5.7 (35.0–76.0)	Adenomyosis; uterine fibroids	Hysterectomy
(86)	Lebanon	1979–1981	Retrospective	Women who have undergone gynecological laparoscopy in a University hospital	862	31.5 ± 4.7 (17.0–52.0)	Endometriosis	Laparoscopy
(87)	Lebanon	Not reported	Retrospective	Electronic records screened for women presenting with hirsutism to the endocrinology clinic	146	22.0 ± 5.1 (Not reported)	PCOS	Rotterdam criteria
(88)	Middle Eastern women in Sweden	1990–2004	Retrospective	The total population register, Swedish national hospital discharge register, and the national cause of death register (only middle eastern population was extracted)	46,384	Not reported (20.0–41.0)	Endometriosis	Laparoscopy
(89)	Oman	2006–2010	Retrospective	Electronic records screened for premenopausal women presenting to gynecology outpatients in a University hospital	3,644	24.2 ± 5.4 (15.0–45.0)	PCOS	Rotterdam criteria
(90)	Oman	2007–2009	Retrospective	Women attending a hospital	3,560	Not reported (18.0–45.0)	PCOS	Rotterdam criteria
(91)	Palestine	2011–2012	Cross-sectional	Random sample of students selected from An-Najah National University	137	20.2 ± 1.40 (18.0–24.0)	PCOS	NIH criteria
(92)	Qatar	Not reported	Cross-sectional study	Eligible participants from the Qatar Biobank	720	27.2 ± 2.68 (18.0–40.0)	PCOS	NIH criteria
(93)	Qatar	2011	Cross-sectional study	Random sample of students from University	120	21.0 ± 2.00 (18.0–30.0)	PCOS	NIH criteria
(94)	Saudi Arabia	1990–2002	Retrospective	Women attending a hospital for an ultrasound	1,111	Not reported (15.0–79.0)	Uterine fibroids	Ultrasound
(95)	Saudi Arabia	1995–1999	Retrospective	Women attending a hospital for a hysterectomy	108	49.8 ± 8.2 Not reported	Uterine fibroids	Hysterectomy
(96)	Saudi Arabia	2000–2005	Retrospective	Women presenting with hirsutism to the endocrinology clinic in a University hospital	101	24.5 ± 6.6 (Not reported)	PCOS	Rotterdam criteria
(97)	Saudi Arabia	2008–2013	Retrospective	Women who have undergone gynecological laparoscopy in a University hospital	190	33.8 ± 8.9 (Not reported)	Endometriosis	Laparoscopy
(98)	Saudi Arabia	1990–2002	Retrospective	Women attending a hospital for a hysterectomy	251	Not reported (18.0–60.0)	Uterine fibroids	Hysterectomy
(99)	Turkey	2017–2019	Retrospective	Women attending infertility polyclinics	3,033	Not reported	PCOS	Rotterdam criteria
(100)	Turkey	2009–2014	Retrospective	Women attending a hospital for a hysterectomy due to heavy bleeding	129	49.0 ± 3.1 (Not reported)	Adenomyosis; uterine fibroids	Hysterectomy
(101)	Turkey	2003–2004	Retrospective	Women attending a hospital for a hysterectomy	298	50.0 ± 4.1 (38.0–86.0)	Adenomyosis	Hysterectomy
(6)	Turkey	2009–2010	Cross-sectional study	Female staff of a governmental institute	392	33.0 ± 7.30 (18.0–45.0)	PCOS	Rotterdam/NIH criteria
(102)	UAE	2016	Cross-sectional study	Self-administered questionnaire from random women selected at a University	3,572	24.9 ± 5.2 (18.0–55.0)	Endometriosis	Laparoscopy
(103)	UAE	1990–1994	Retrospective	Women attending an endocrine clinic for hirsutism	102	22.0 ± 5.1 (14.0–37.0)	PCOS	Rotterdam criteria
(104)	UAE	2012–2015	Retrospective	Women who have undergone gynecological laparoscopy in an endometriosis clinic in a hospital	5,881	32.1 ± 5.8 (15.0–59.0)	Endometriosis	Laparoscopy
(105)	UAE	2012	Cross-sectional study	Random sample of students selected from Ras Al Khaimah Medical University	250	19.7 ± 1.68 (18.0–24.0)	PCOS	NIH Criteria
(106)	Yemen	2006–2012	Retrospective	Women attending a hospital for a hysterectomy	2,544	47.6 ± 5.0 (16.0–80.0.0)	Adenomyosis; uterine fibroids	Hysterectomy

*NIH, National Institute of Health; PCOS, polycystic ovary syndrome*.

Of the 47 included studies, 27 received a GRADE score of “very low” and 20 studies received a GRADE score of “low” (Supplementary Table 1). The summary of the quality assessment for the studies included in the meta-analysis for each study outcome (*N* = 11; grouped based on multiple diagnostic criteria, sampling method, and definition of patient group prevalence estimation provided by each study, see Methods) also showed GRADE scores of “very low” and “low” (Table 3).

**Table 3:** Summary of the quality assessment of the studies reporting the outcomes of interest included in the meta-analyses.

**Study outcome**	**No. of studies**	**Grade score**	**Study design**	**Risk of bias**	**Inconsistency**	**Indirectness**	**Imprecision**	**Publication bias**	**Uplifting factors**
**Polycystic ovary syndrome**									
Population-based study: NIH criteria	12	Very low	2; non-RCT	−1; Six studies with high risk of bias (unrepresentative sample of population; did not specify recruitment strategy)	−1; high heterogeneity due to inconsistency in recruitment strategy and target population	0; all studies demonstrated directness of evidence	−1; two studies with imprecision (sample size not statistically significant)	0; none	2; Four studies with representative sample and similar direction of effect; four studies with large sample size
Population-based study: Rotterdam criteria	6	Low	2; non-RCT	0; five studies with low risk of bias (all samples were selected from the same country)	−1; high heterogeneity due to inconsistency in recruitment strategy and target population	0; all studies demonstrated directness of evidence	0; all studies demonstrated precision in sample	0; None	1; three studies with representative sample and similar direction of effect; two studies with large sample size
Hospital-based study: any indication via Rotterdam criteria	3	Low	2; non-RCT	0; two studies with low risk of bias (retrospective studies that identifies prevalence within a defined group)	0; no heterogeneity	0; all studies demonstrated directness of evidence	−1; one study with imprecision (sample size not statistically significant)	0; none	1; two studies with large sample size, adequate disease ascertainment
Hospital-based study: infertility via Rotterdam criteria	5	Very low	2; non-RCT	−1; three studies with high risk of bias (unrepresentative of control group and whole study population)	−1; high heterogeneity due to inconsistency in recruitment strategy and target population with inadequate sample ascertainment	0; all studies demonstrated directness of evidence	0; all studies demonstrated precision in sample	0; none	1; three studies with large sample size
**Endometriosis**									
Population-based study	4	Very low	2; non-RCT	−1; two studies with high risk of bias (unrepresentative of control group or whole population; self-reported outcomes)	−1; high heterogeneity due to inconsistency in recruitment strategy and target population	0; all studies demonstrated directness of evidence	−1; two studies with imprecision (recruited a younger sample group)	0; none	2; two studies with representative sample and similar direction of effect
Hospital-based study: any indication	4	Very low	2; non-RCT	0; two studies with low risk of bias (retrospective studies that identifies prevalence within a defined group)	−1; high heterogeneity due to inconsistency in recruitment strategy and target population	−1; two studies demonstrated indirectness of evidence (did not report total sample size in manuscript)	−1; one study with imprecision (sample size not statistically significant)	0; none	1; two studies with large sample size, adequate disease ascertainment
Hospital-based study: infertility	3	Low	2; non-RCT	0; two studies with low risk of bias (unrepresentative of population)	−1; high heterogeneity due to inconsistency in recruitment strategy and target population	0; all studies demonstrated directness of evidence	0; all studies demonstrated precision in sample	0; None	1; two studies with large sample size, adequate disease ascertainment
**Uterine fibroids**									
Hospital-based study: any indication	6	Low	2; non-RCT	−1; five studies with high risk of bias (unrepresentative of control group and whole study population; retrospective studies)	−1; high heterogeneity due to inconsistency in recruitment strategy and target population	0; all studies demonstrated directness of evidence	0; all studies demonstrated precision in sample	0; none	2; two studies reported adequate disease ascertainment and high sample size
Hospital-based study: abnormal uterine bleeding	2	Very low	2; non-RCT	−1; two studies with high risk of bias (unrepresentative of control group and whole study population; retrospective studies)	−1; high heterogeneity due to inconsistency in recruitment strategy and target population	0; all studies demonstrated directness of evidence	−1; two studies with imprecision (sample size not statistically significant)	0; none	1; one study reported adequate disease ascertainment and significant sample size
**Adenomyosis**									
Hospital-based study: any indication	4	Low	2; non-RCT	−1; three studies with high risk of bias (unrepresentative of control group and whole study population; retrospective studies)	−1; high heterogeneity due to inconsistency in recruitment strategy and target population	0; all studies demonstrated directness of evidence	0; all studies demonstrated precision in sample	0; none	2; two studies reported adequate disease ascertainment and significant sample size
Hospital-based study: abnormal uterine bleeding	2	Very low	2; non-RCT	−1; two studies with high risk of bias (unrepresentative of control group and whole study population; retrospective studies)	0; no heterogeneity	0; all studies demonstrated directness of evidence	−1; two studies with imprecision (sample size not statistically significant)	0; none	1; one study reported adequate disease ascertainment and significant sample size

### Polycystic Ovary Syndrome

**Figure 2:**
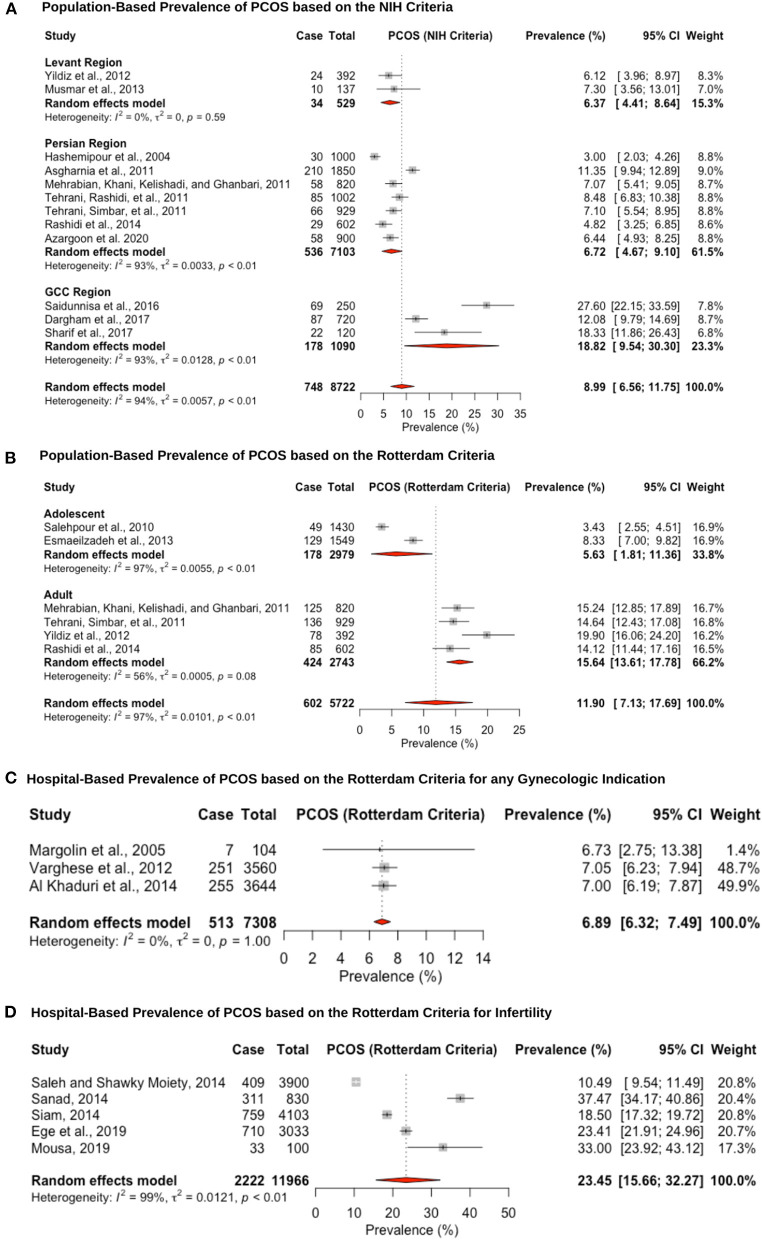
Forest plot of the pooled prevalence estimates of PCOS: **(A)** population-based study based on the NIH criteria, **(B)** population-based study based on the Rotterdam criteria, **(C)** hospital-based study (obstetrics and gynecology department) based on the Rotterdam criteria, and **(D)** hospital-based study (presenting with infertility) based on the Rotterdam criteria.

Twenty-six studies reported prevalence estimates of PCOS, with eight reporting NIH criteria-based prevalence, 15 Rotterdam criteria-based prevalence, and three studies utilizing both the NIH and Rotterdam criteria (Figure 2). The population-based pooled prevalence of PCOS according to NIH criteria was 8.9% (95% CI: 6.5–11.7; range: 3.0–27.6%) (Figure 2A). Due to high heterogeneity (*I*^2^ = 94.0%) observed among the studies, results are presented from a random-effects model. Pooled prevalence of PCOS in the Levant region and Persian region was estimated at the lower range of the scale with 6.3% (95% CI: 4.4–8.6; range: 6.1–7.3%) (6, 91) and 6.7% (95% CI: 4.6–9.1; range: 3.0–11.3%) (69, 71, 74, 75, 77–79), respectively, whereas prevalence estimates in the Gulf Arab states were 18.8% (95% CI: 9.5–30.3; range: 12.0–27.6%) (92, 93, 105). The population-based pooled prevalence according to the Rotterdam criteria was 11.9% (95% CI: 7.1–17.6; range: 3.4–19.9%) but only included studies from Iran and Turkey, from the Persian and Levant regions (Figure 2B). Among post-menarche adolescent women (<18 years of age), the pooled prevalence was 5.6% (95% CI: 1.8–11.3; range: 3.0–8.3%) (76, 107), whereas among pre-menopausal adult women (>18 years of age), it was much higher, at 15.6% (95% CI: 13.6–17.7; range: 14.1–19.9%) (6, 74, 75, 78).

The hospital-based prevalence studies of PCOS were categorized into (i) women presenting to obstetrics and gynecology clinics with any indication: 6.8% (95% CI: 6.3–7.4; range: 6.7–7.0%) (Figure 2C) and (ii) infertile women: 23.4% (95% CI: 15.6–32.2; range: 10.4–37.4) (Figure 2D). The three hospital-based studies estimated lower prevalence rates, but one of the studies only recruited post-menopausal women (6.7%, 95% CI: 3.3–13.2) (83), and the other two analyzed medical records retrospectively (7.0%, 95% CI: 6.2–7.9 and 7.0%, 95% CI: 6.1–7.8) (89, 90). The high heterogeneity (*I*^2^ = 99%) that was observed for the infertile pooled prevalence estimate is likely due to the fact that two of the studies were recruiting participants from a specialized infertility clinic (10.4%, 95% CI: 9.5–11.44 and 23.4%, 95% CI: 21.9–24.9) (63, 99), whereas the other three studies were recruiting infertile women that were visiting the obstetrics and gynecology department in a University hospital (18.5%, 95% CI: 17.3–19.7; 37.4%, 95% CI: 34.1–40.8 and 33.0%, 95% CI: 23.9–43.1) (64, 65, 80). Hence, the former group may be women with severe PCOS phenotype that require specialized fertility services and assistive reproductive technology, whereas the latter group may be women who require fertility medical treatment. Since hyperandrogenism is part of the Rotterdam criteria, and hirsutism is caused by increased androgenicity in the pilosebaceous gland, the pooled prevalence within the hirsute patients diagnosed with PCOS was very large, at 70.2% (95% CI: 57.7–81.7; range: 62.5–82.1%; *I*^2^ = 96%) (67, 87, 96, 103, 108).

### Endometriosis

**Figure 3:**
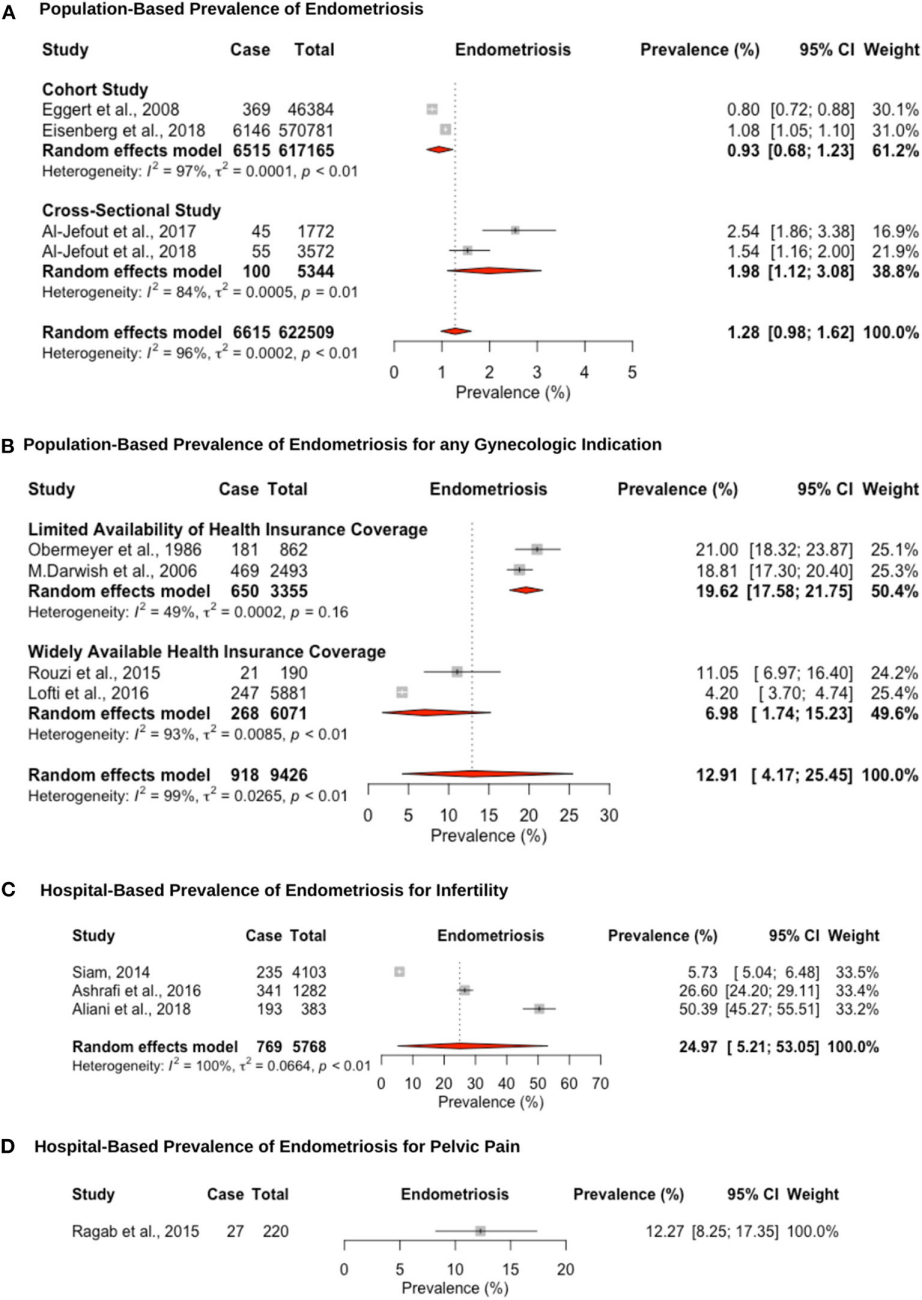
Forest plot of the pooled prevalence estimate of surgically confirmed endometriosis: **(A)** population-based study, **(B)** hospital-based study (undergoing laparoscopy for any reason), **(C)** hospital-based study (presenting with infertility), and **(D)** hospital-based study (presenting with pelvic pain).

Twelve studies reported prevalence estimates of endometriosis diagnosed via laparoscopic surgical visualization, and four out of these 12 reported additional histological confirmation (Figure 3). The pooled prevalence for surgically confirmed endometriosis in population studies, which were validated through surgical records, was estimated at 1.2% (95% CI: 0.9–1.6, range: 0.8–2.4%) (Figure 3A). Two of these studies retrospectively extracted data from large-scale population-based databases in Israel (82) and Middle Eastern women living in Sweden (88). Two further cross-sectional studies in Jordan (84) and the U.A.E. (102) assessed women attending universities who could be affected but given the younger age may not have undergone laparoscopic intervention or sought medical help.

The hospital-based prevalence for studies of endometriosis among women undergoing laparoscopy for unspecified indication was 12.9% (95% CI: 4.1–25.4, range: 4.2–21.0%). However, differences in prevalence estimates were observed based on the availability of healthcare insurance coverage (Figure 3B). Despite the importance of healthcare insurance coverage to provide cost-effective, quality healthcare services, most of the region did not provide or implement insurance coverage for the citizens and residents until the World Health Assembly passed a resolution in 2005 (30, 109, 110). Articles from countries that were published before 2006 had limited healthcare insurance coverage with a pooled prevalence estimate of 19.6% (95% CI: 17.5–21.7; range: 18.8–21.0%) (61, 86), as opposed to a prevalence estimate of 6.9% (95% CI: 1.7–15.2; range: 4.2–11.0%) (84, 102) for articles in countries published after 2007 with highly available private healthcare insurance coverage. Wider healthcare insurance coverage may reflect the lower prevalence estimate for surgically confirmed endometriosis, due to more accessibility to surgical procedures (111). The pooled hospital-based prevalence of endometriosis among infertile women undergoing laparoscopy was higher than for any indication, at 24.9% (95% CI: 5.2–53.0), although study estimates varied widely (range: 5.7–50.3%) (65, 66, 68) (Figure 3C). One large prospective study in Egypt (*N* = 2,493) showed that infertility is the primary reason for laparoscopic surgery in 80.9% of surgeries, followed by chronic pelvic pain in 12.2% and other indications in 14.5%. Endometriosis was diagnosed in 38.8, 46.6, and 14.5% of these three groups, respectively (61). This suggests that patients are more likely to seek medical treatment due to infertility compared to pelvic pain or other indications, which may be due to limited access of unmarried women to gynecology clinics. The hospital-based prevalence of endometriosis among women undergoing laparoscopy for pelvic pain was only estimated in a single study, at 12.2% (95% CI: 8.2–17.3) (62) (Figure 3D). The prevalence of endometriosis is likely overestimated among women undergoing surgery for pain symptoms and/or infertility; hence, there is an inherent selection bias in these groups compared to the general population.

Endometriosis was staged laparoscopically according to the four stages of the American Society of Reproductive Medicine (ASRM) staging system in 4 out of 12 studies (61, 68, 104, 112). The laparoscopic presentation of stage I/II vs. stage III/IV endometriosis was noted in one study (66). The endometriosis stage was not mentioned in eight of the 12 studies. Pooled prevalence of endometriosis for patients undergoing laparoscopy for any indication, according to the ASRM staging system, was 21.7% (95% CI: 13.8–30.8, *I*^2^ = 92.8%) for stage I, 34.1% (95% CI: 25.3–43.4, *I*^2^ = 73.7%) for stage II, 26.1% (95% CI: 14.9–39.1, *I*^2^ = 96.1%) for stage III, and 15.3% (95% CI: 8.03–24.3) for stage IV. Only one study reported the prevalence of endometriosis, according to ASRM criteria, for patients undergoing laparoscopy for infertility: 15.3% (52/341) for stage I, 24.9% (85/341) for stage II, 32.5% (111/341) for stage III, and 24.6% (84/341) for stage IV (68), and one study for patients undergoing laparoscopy for pelvic pain: 44.4% (12/27) for stage I, 25.9% (7/27) for stage II, and 29.6% (8/27) for stage III (62). Stage was not correlated with pelvic pain or infertility, as reported in previous studies (113).

### Uterine Fibroids

**Figure 4:**
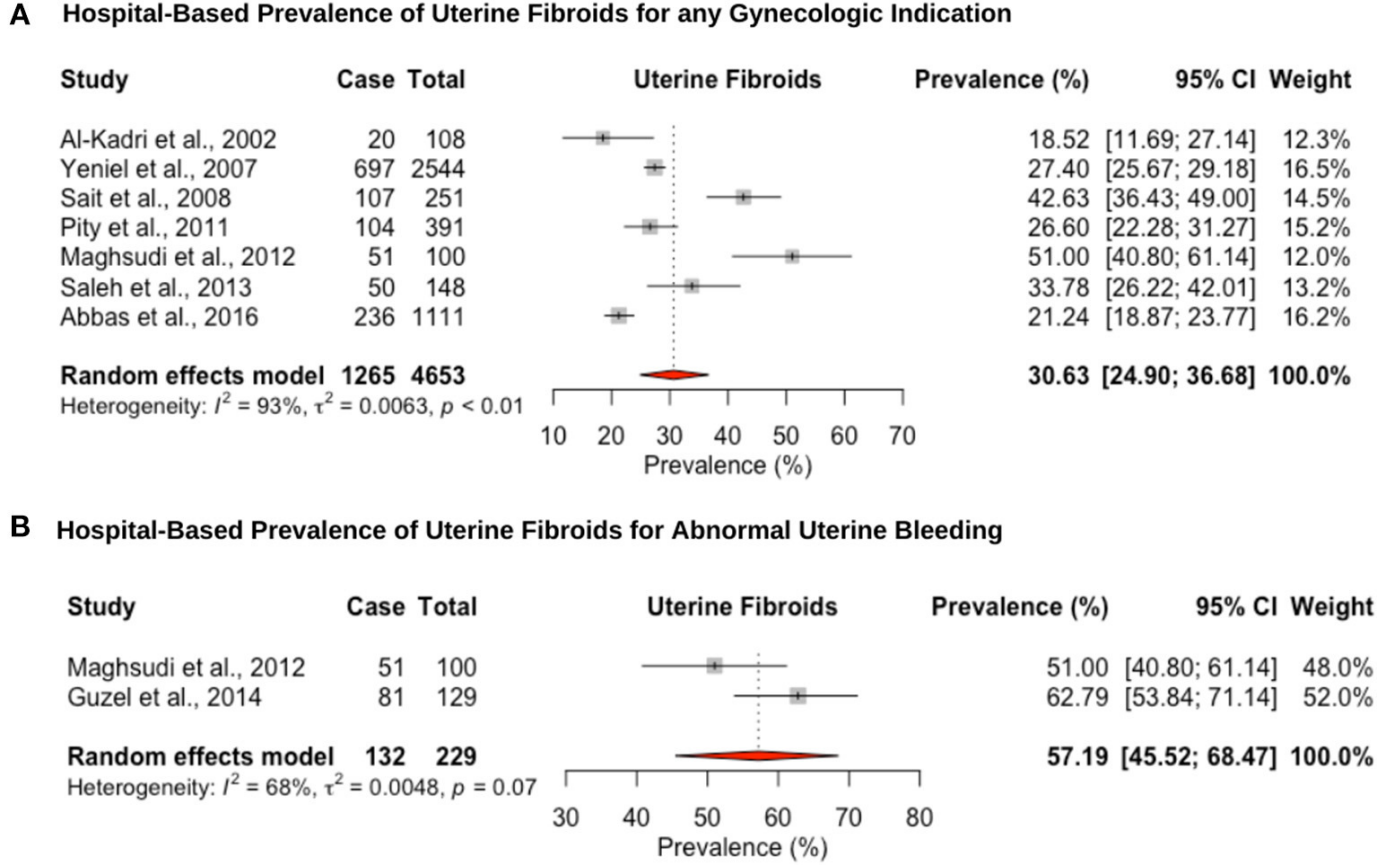
Forest plot of the pooled prevalence estimate of hysterectomy-confirmed uterine fibroids: **(A)** hospital-based study (undergoing hysteroscopy in a period of time) and **(B)** hospital-based study (presenting with abnormal uterine bleeding).

Eight hospital-based studies estimated the prevalence of uterine fibroids by retrospectively screening electronic medical records of women (Figure 4). Seven studies utilized hysteroscopy as the diagnostic tool, and only one study utilized imaging and clinical assessment to diagnose uterine fibroids. The pooled hospital-based prevalence of uterine fibroids in women undergoing hysterectomy or ultrasound was 30.6% (95% CI: 24.9–36.6; range: 18.5–42.6%) for any indication (Figure 4A). The pooled hospital-based prevalence of uterine fibroids in women undergoing hysterectomy for abnormal uterine bleeding was 57.1% (95% CI: 45.5–68.4; range: 51.0–62.7%) (Figure 4B), while the prevalence of uterine fibroids diagnosed by ultrasound was 21.2% (95% CI: 18.8–23.7).

### Adenomyosis

**Figure 5:**
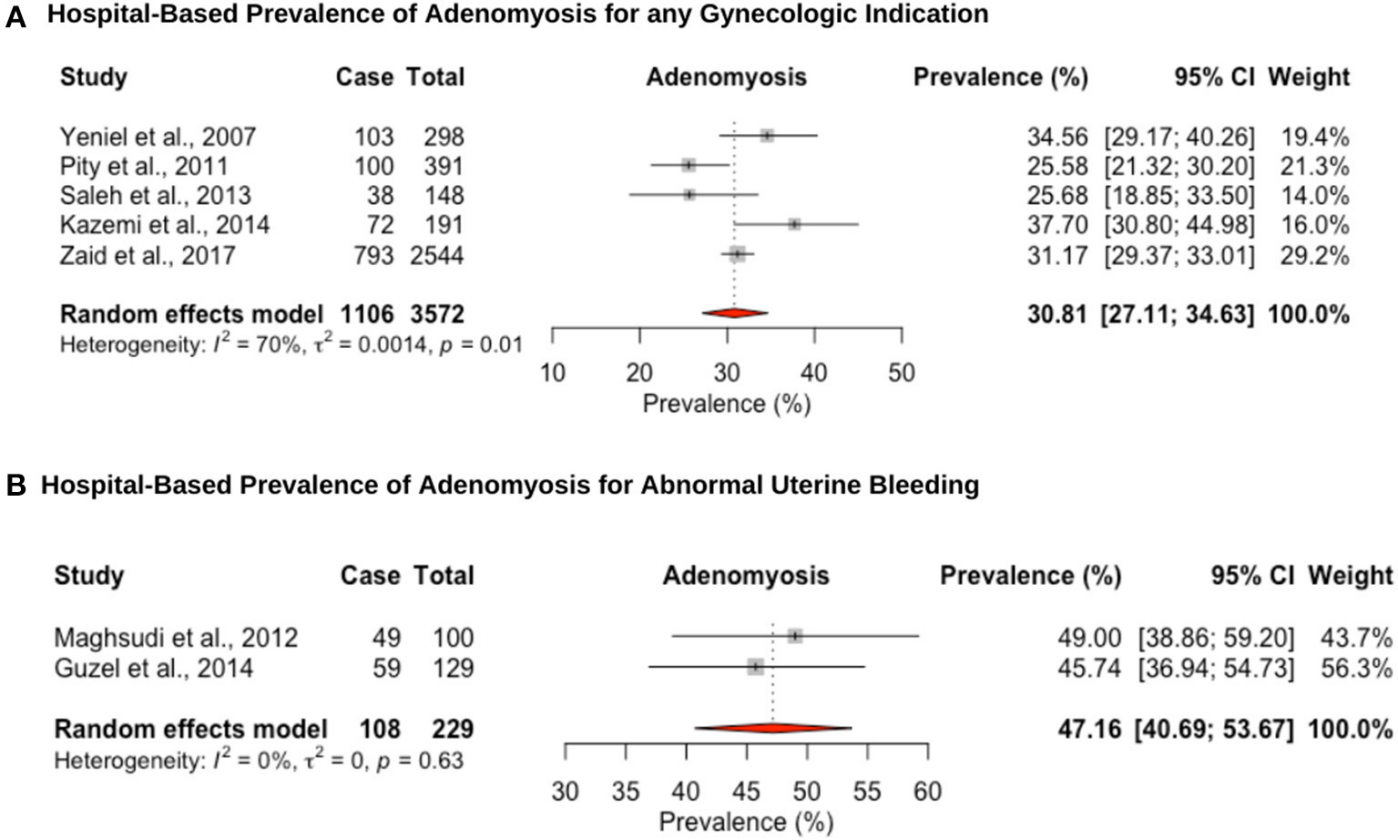
Forest plot of the pooled prevalence estimate of hysterectomy-confirmed adenomyosis: **(A)** hospital-based study (undergoing hysteroscopy in a period of time) and **(B)** hospital-based study (presenting with abnormal uterine bleeding).

Five hospital-based studies reported the prevalence of adenomyosis by retrospectively screening electronic medical records of women attending a hospital for a hysterectomy (Figure 5). The hospital-based prevalence of adenomyosis in women undergoing hysterectomy for any indication was 30.8% (95% CI: 27.1–34.6, range: 25.5–37.7%) (Figure 5A) and 47.2% (95% CI: 40.7–53.7, range: 45.7–49.0%) for heavy menstrual bleeding (Figure 5B). Women diagnosed with adenomyosis often have other associated gynecological diseases, such as endometriosis or uterine fibroids, which complicates differential diagnosis and attribution of symptoms. In addition, no study provided an imaging-based diagnosis that could provide a more representative population-based estimate. While the gold standard for diagnosis has been histopathologic examination of the uterus after hysterectomy, the prevalence may be underestimated due to missed adenomyosis from cases that are not managed by hysterectomy (114).

## Discussion

### Main Findings

We conducted the first systematic review and meta-analysis of pooled prevalence of PCOS, endometriosis, uterine fibroids, and adenomyosis in women from the Middle East, a region representing 6.8% of the world's population but underserved in clinical science (28–31). Much of the scientific research featured poorly designed studies and methodological and statistical heterogeneity. The region lacks reliable, population-based data on the major causes of women's health diseases due to limited reporting regulations and lack of national registries (29, 31).

Our findings indicate that the population-based prevalence of PCOS according to NIH and Rotterdam diagnostic criteria in the Middle Eastern populations is estimated at 8.9% (95% CI: 6.5–11.7) and 11.9% (95% CI: 7.1–17.6), respectively, which are similar to the estimated global prevalence (5–9% and 10–20%, respectively) (2). However, among women from the Gulf Region, there was a substantially higher prevalence of PCOS (18.8%; 95% CI: 9.5–30.3) in comparison to women from the Levant (6.3%; 95% CI: 4.4–8.6) and Persian Region (6.7%; 95% CI: 4.6–9.1), based on the NIH criteria. Obesity rates in the Arab Gulf countries exceed those of the remaining Middle Eastern region, with a gender disparity among women, for all age groups (115), where the prevalence of overweight and obesity among adults is 35–48 and 24–40%, respectively (116). Obesity is a common clinical feature in women affected by PCOS, due to its association to the onset of oligomenorrhea and hyperandrogenism (117). Comorbid conditions of PCOS, such as hyperinsulinemia, type 2 diabetes mellitus, cardiovascular disease, and dyslipidemia, have also been reported to have the highest prevalence in the Arab Gulf countries (118–120). Hence, it is likely that the prevalence differences of PCOS between the regions are at least in part due to the pathogenetic role on obesity in the subsequent development of PCOS (117). Analysis of a genome-wide association study (GWAS) on PCOS demonstrated that the relative severity of the PCOS phenotypes, in particular severe hirsute-hyperandrogenism, may result from different combinations of SNPs more commonly present in women of Middle Eastern origin. This may be a basis for the higher prevalence of PCOS that is dominated by obesity, high blood pressure, diabetes mellitus, and hirsutism (121, 122). Genetic influences in populations of diverse origin are important to study alongside environmental influences, to understand the effect of their interaction in causing different PCOS phenotypes (123). Additionally, high heterogeneity was reported due to variability of age group of the participants among studies, where post-menarche adolescent women and post-menopausal women reported a lower prevalence of PCOS compared to pre-menopausal adult women. Adolescents with PCOS manifest similar clinical, metabolic, and endocrine features to adult women (124); hence, the significant difference could be attributed to delayed diagnosis of the condition among young, single women who are unable to access reproductive health care due the hegemony of traditions that may prevent them from discussing their symptoms and seeking care.

Prevalence for surgically confirmed endometriosis (12.9% among women undergoing laparoscopy for any indication; 24.% among infertile women; 12.2% among women hospitalized for pelvic pain; 1.28% in a population-based setting) was similar to European ancestry populations (5–10% among women undergoing laparoscopy; 5–50% among infertile women; 5–21% among women hospitalized for pelvic pain; 2–43% among asymptomatic women seeking tubal ligation) (4, 17, 125). However, the reliance on surgical confirmation of endometriosis, access to clinics that is mostly limited to married women, and consequent selection biases affect these estimates, as is demonstrated by the heterogeneity estimates in our meta-analyses. As the main presenting symptomatology of endometriosis and mode of diagnosis are both influenced by psychosocial factors, women of different cultural backgrounds may present with different clinical phenotypes. Further epidemiological studies must be conducted among women of different populations, to explore the diagnostic pathways for women with endometriosis and be able to obtain an accurate prevalence estimate for the population. In addition, given that endometriosis has an estimated heritability of ~50% and the region has an estimated consanguinity rate of 20–50%, increased prevalence of endometriosis may be mediated by numerous deleterious recessive alleles due to increased homozygosity levels (126–128). Most GWAS datasets have been generated from women of European ancestry, with only two in women of East Asian ancestry. It is important to define the extent of the effects of specific risk alleles in different ethnic groups and conduct trans-ethnic mapping to restrict signal to causal variants of endometriosis (129–131).

The hospital-based pooled prevalence of uterine fibroids for women was estimated at 28.2% (95% CI: 20.4–36.7), vs. 57.1% (95% CI: 45.5–68.4) among women with heavy menstrual bleeding, which are on the high end of the estimated global prevalence 5–30% (3) and 37–59% (132), respectively. Detection bias may explain the overestimation of uterine fibroid prevalence as nearly all studies were from women presenting for hysterectomy. Considering the consistently observed greater risk of uterine fibroids among black women, it is important to conduct further more robust epidemiological and genetic studies in women of Middle Eastern origin, to investigate whether they also exhibit a higher prevalence and whether this is associated with certain risk factors (3, 132, 133). The hospital-based prevalence of adenomyosis for women undergoing hysterectomy was high (any indication: 31.7% [95% CI: 20.6–43.9; for heavy menstrual bleeding: 47.2% (95% CI: 40.7–53.7)], but within the range previously reported for studies assessing prevalence at hysterectomy (14–57%) (21). The restriction of hospital-based studies to women undergoing hysterectomy for adenomyosis likely underestimates the prevalence as tissue may not have been routinely investigated for the condition. In addition, prevalence studies of uterine fibroids and adenomyosis all recruited multiparous women in their fourth and fifth decades of life, making comparison of estimates with global figures difficult. The lack of well-conducted, population-based studies limits the understanding of the public health impact of these conditions and prioritize diagnostic and management pathways.

### Limitations and Strengths

This is the first systematic review of the prevalence of common gynecological conditions in Middle Eastern women. Study and reporting quality were generally low; however, the moderate/high heterogeneity that were present in the pooled data was reduced after completing subgroup analysis using relevant variables to limit bias. Potential explanations of residual heterogeneity among the studies are likely due to differences in study populations, sampling schemes, and study designs (case definition and ascertainment). Most of the studies selected women through convenience sampling, from hospitals, which may either overestimate the prevalence of the diseases or underestimate the disease risk due to missed asymptomatic cases. Prevalence estimates from retrospectively screened medical records are potentially affected by selection and information biases, as well as methodological issues, such as a lack of specific inclusion and exclusion criteria and data abstraction protocols. Beyond the limitations of the existing literature, there are fundamental issues with the diagnostic method used for the gynecological conditions, which hinders derivation of true population prevalence. The heterogeneous nature of PCOS in terms of its definition, diagnostic criteria, and ethnic variability likely leads to variable PCOS prevalence estimates in different populations. The lack of a non-invasive diagnostic modality for endometriosis creates diagnostic bias for those that are unable to access surgical evaluation. In addition, much of the information available on uterine fibroids and adenomyosis prevalence came from hospital-based studies that lacked imaging and symptomatology-based diagnosis and were not representative of the general population.

Social and cultural factors play a role in diagnostic bias. Given that women in the region will more likely consult a physician for fertility-related issues, as opposed to pain or menstrual irregularities (61), the studies conducted in a hospital-based setting are based on highly selected populations that cannot be generalized broadly. In addition, gynecological conditions and associated clinical phenotypes may present differently in various populations. Given the lack of epidemiological studies in the region focusing on symptomatology and phenotypes, the standard clinical definitions based on mainly Western population health data may not adequately capture the symptom presence and severity affecting diagnostic pathways of these heterogeneous conditions (134, 135). In fact, treatment regimens and responses vary widely between regions and among ethnic groups (136). There is an underrepresentation of studies from low- and middle-income regions in the Middle East that have been subject to war and political instability (Iraq, Syria, Libya, Sudan, Lebanon, Yemen, and Palestine), as opposed to higher-income regions, such as the Gulf countries, Turkey, and Egypt, where women have greater access to health care and may be more intensively screened.

This review highlights the importance of investigating gynecological health in the Middle East, and the need for well-designed studies that address both prevalence and the impact of gynecological condition on women's lives. An extensive search was conducted in two languages, to avoid missing any relevant information. We performed a broad search, including manuscripts from gray literature. Potential sources of bias were identified, and significant clinical and statistical heterogeneity was discussed to indicate considerable variability in the data due to residual confounding and study design. It is essential to prioritize improved and unbiased quantification of gynecological condition prevalence and incidence extrapolation, to improve generalizability and improve public health focus.

Further epidemiological studies must be conducted to investigate symptomatology and risk factors in women of this region, to deliver more culturally sensitive healthcare (137). Specific areas in need of attention include improving the clinical definition, diagnostic techniques, and treatment methods to provide the best practice for this diverse patient population. More generally, given the potential effects of these common chronic conditions on personal lives, economies, healthcare systems, and global community, it must be a public health priority to advance our understanding of the complex pathways leading to diagnosis, develop targeted prevention, and provide early detection guidelines in different populations (138–143). Key to this endeavor is advancing our understanding of how genetic and non-genetic factors interplay to disease predisposition and mechanisms in different populations (144).

## Conclusion

To our knowledge, this is the first systematic review study to investigate the prevalence of common gynecological diseases in women in Middle Eastern populations, highlighting a paucity in reliable data. With 200 million women in the region, the importance of conducting more research in the area of women's health is clear and is needed to serve as a benchmark for evaluation of future research activities and comparative purposes with Middle Eastern immigrants and other populations. A gynecology clinic in the Middle East remains the realm mostly of married women. Young women may suffer from chronic pain and menstrual irregularities, but the hegemony of traditions may prevent them from discussing these and thus prolong suffering. To continue to decrease health disparities, researchers should acknowledge the limitations in scientific research that investigate the health disparities in women of Middle Eastern region. Robust data will urge policymakers in the Middle East to invest in health information systems to better identify risk factors for gynecology diseases, help set targets for policymaking, impact public awareness, and enable educational and evaluation strategies.

## Data Availability Statement

The original contributions presented in the study are included in the article/Supplementary Material, further inquiries can be directed to the corresponding author/s.

## Author Contributions

MM and NR were involved in all parts of the study: conception, study design, acquisition of data, information extraction, quality assessment, analysis, meta-analysis plots, interpretation, drafting of manuscript, and final approval. KZ and CB were responsible for conception, study design, analysis methods, interpretation, drafting of manuscript, and final approval. SK identified the search terms, developed the search strategies, conducted the search on each database to find relevant studies, reviewed the literature search method section of the manuscript, and did the final approval. MA-J, HA, CL, and SM were involved in study design, interpretation, reviewing of the manuscript, and final approval. All authors contributed to the article and approved the submitted version.

## Conflict of Interest

SM has been a member of advisory/scientific boards for AbbVie. CB reports grant from Bayer AG, others from AbbVie Inc., grants from Volition Rx, grants from MDNA Life Sciences, grants from Roche Diagnostics Inc., nonfinancial support from Population Diagnostics Ltd, others from ObsEva, and others from Flo Health, outside the submitted work. KZ reports grants from Bayer Healthcare, MDNA Life Sciences, Roche Diagnostics Inc., Volition Rx, and Evotec (Lab282 - Partnership program with Oxford University), nonfinancial support from AbbVie Ltd, all outside the submitted work, and is a Board member (Secretary) of the World Endometriosis Society and World Endometriosis Research Foundation. The remaining authors declare that the research was conducted in the absence of any commercial or financial relationships that could be construed as a potential conflict of interest.
