# Supplementary material for: Prevalence of Common Gynecological Conditions in the Middle East: Systematic Review and Meta-Analysis
Source: Front Reprod Health. 2021 Apr 6;3:661360. doi: 10.3389/frph.2021.661360 (PMC9580651; doi:10.3389/frph.2021.661360)
Supplement: Supplementary file 1 [file Data_Sheet_1.PDF]

Supplementary Material

1 Supplementary Text 1

Search Platform - Wolters Kluwer Ovid

Database: Medline (Ovid MEDLINE Epub Ahead of Print, In-Process & Other Non-Indexed Citations, Ovid MEDLINE Daily and Ovid MEDLINE) 1946 to present; PsycINFO 1806 to present (via OVID); Global Health 1973 to 2018 Week 50 (via Ovid); Embase 1974 to present (via OVID)

Search date: 14 February 2021

{Gynecological Disorders/ Reproductive Health/ Menstrual Disorders/ Menstruation/ Menarche/ Dysmenorrhea/ "Endometriosis".mh./ endometrio$ or dysmenorrh$ or adenomyo$).ti,ab,id./ "Polycystic Ovary Syndrome".mh./ (polycystic adj1 (ovarian or ovary) adj1 syndrome).ti,ab,id./ PCOS.ti,ab,id./"Leiomyoma".mh/ (leiomyoma or fibroid$ or leiomyomata).ti,ab,id./ exp Sexually Transmitted Diseases/ (sexual$ adj1 transmit$ adj1 (infection$ or disease$)).ti,ab,id./ (STD or STDs or STI or STIs).ti,ab,id./ "Pelvic Inflammatory Disease".mh./ ((pelvic or pelvis) adj1 inflammatory adj1 disease$).ti,ab,id./PID.ti,ab,id./ (adnexal adj1 mass$).ti,ab,id.} AND {["Genetic Predisposition to Disease".mh./ Genetics/ Genes/ Genotypes/ Alleles/ "Genetic Loci".mh./ Predisposition/ Polymorphism/ (genotyp$ or variant$ or allele$ or allelomorph$ or locus or loci or SNP or SNPs or inherit$ or trait$ or mutation$ or epigenetic$ or gene$ or genome$).ti,ab,id./ (single adj1 nucleotide adj1 polymorphism$).ti,ab,id./ "candidate gene".ti,ab,id./ "Gene Frequency".mh./ "Genetic Association Studies".mh./ Genome/ associat$.ti,ab,id.] OR [Epidemiology/ "Epidemiologic Factors".mh./ "Descriptive Epidemiology".mh./ (epidemiolog$ or occurrence$ or frequency or environment$).ti,ab,id./ "Prevalence".mh./ prevalence.ti,ab,id/ "Incidence".mh./ incidence.ti,ab,id./ "Rate".mh./ Rate.ti,ab,id./ "Frequency".mh./ Frequency.ti,ab,id./ Risk factors/ "Risk".mh./ risk.ti,ab,id./ Comorbidity/ (co adj1 morbidit$).ti,ab,id.]} AND { (Jordan or Iran or Iraq or Israel or Turkey or Syria or "Saudi" or "Saudi Arabia" or Kuwait or Yemen or Qatar or Bahrain or Oman or Lebanon OR Sudan or Tunisia or Algeria or Libya or Morocco or Somalia or Cyprus or Egypt or Emirates).ti,ab,id./ (Jordanian or Iraqi or Irani or Iranian or Jewish or Turkish or Syrian or Kuwaiti or Yemeni or Qatari or Bahraini or Omani or Lebanese or Sudanese or Tunisian or Algerian or Libyan or Moroccan or Somali or Somalian or Cypriot or Egyptian or Emirati or Arab).ti,ab,id./ Middle East".mH/ "Africa, Northern".mh./ North Africa/ Arab Countries}

2 Supplementary Table

Supplementary Table 1. Quality Assessment for each study, as per the GRADE criteria.

Study	Risk of bias	Inconsistency	Indirectness	Imprecision	Publication bias	Overall certainty of evidence
(Al Khaduri et al., 2014)	None	Not Serious	Not Serious	Not Serious	Not Serious	⊕⊕◯◯ Low
(Al-Ruhaily et al., 2008)	None	Not Serious	Not Serious	Serious<br>*No control group to measure hormone imbalance<br>*No adjustment to confounding	Not Serious	⊕◯◯◯ Very Low
(Ansarin et al., 2007)	None	Not Serious	Not Serious	Not Serious	Not Serious	⊕⊕◯◯ Low
(Asgharnia et al., 2011)	None	Not Serious	Serious<br>*Not representative of the population (only high school students)	Serious<br>*Confidence Interval and effect size was not calculated and recorded<br>*No adjustment to confounding	Not Serious	⊕◯◯◯ Very Low
(Azargoon et al., 2020)	None	Not Serious	Serious<br>*Not representative of the population (only high school students)	Serious<br>*Confidence Interval and effect size was not calculated and recorded<br>*No adjustment to confounding	Not Serious	⊕◯◯◯ Very Low
(Dargham et al., 2017)	None	Not Serious	Not Serious	Not Serious	Not Serious	⊕⊕◯◯ Low
(Ege et al., 2020)	None	Not Serious	Not Serious	Serious<br>*Mean age not reported<br>*Confidence Interval and effect size was not calculated and recorded<br>*No adjustment to confounding	Not Serious	⊕◯◯◯ Very Low
(Esmaeilzadeh et al., 2013)	None	Not Serious	Serious	Not Serious	Not Serious	⊕◯◯◯ Very Low

			*Not representative of the population (only women from Infertility Clinic)	*Small sample size (N<100)		
(Gatee et al., 1996)	None	Not Serious	Not Serious	Not Serious	Not Serious	⊕⊕◯◯ Low
(Hashemipour Et Al., 2004)	None	Not Serious	Not Serious	Not Serious	Not Serious	⊕⊕◯◯ Low
(Margolin et al., 2005)	None	Not Serious	Not Serious	Serious<br>*No adjustment to confounding	Not Serious	⊕◯◯◯ Very Low
(Mehrabian, Khani, Kelishadi, and Ghanbari, 2011)	None	Not Serious	Not Serious	Serious<br>*No adjustment to confounding	Not Serious	⊕◯◯◯ Very Low
(Musmar et al., 2013)	None	Not Serious	Not Serious	Serious<br>*No adjustment to confounding	Not Serious	⊕◯◯◯ Very Low
(Rashidi et al., 2014)	None	Not Serious	Not Serious	Not Serious	Not Serious	⊕⊕◯◯ Low
(RS and MP, 2014)	None	Not Serious	Not Serious	Serious<br>*No adjustment to confounding	Not Serious	⊕◯◯◯ Very Low
(Saidunnisa et al., 2016)	None	Not Serious	Not Serious	Very Serious<br>*Confidence Interval and effect size was not calculated and recorded<br>*No adjustment to confounding<br>*Does not report control group outcome and associated variables	Not Serious	⊕◯◯◯ Very Low
(Saleh and Shawky Moiety, 2014)	None	Not Serious	Serious<br>*Not representative of the population (only women with infertility)	Serious<br>*No adjustment to confounding	Not Serious	⊕◯◯◯ Very Low
(Salehpour et al., 2011)	None	Not Serious	Not Serious	Not Serious	Not Serious	⊕⊕◯◯ Low

(Sanad, 2014)	None	Not Serious	Serious<br>*Not representative of the population (only infertile group)	Serious<br>*Confidence Interval and effect size was not calculated and recorded<br>*No adjustment to confounding	Not Serious	⊕◯◯◯ Very Low
(Sharif et al., 2017)	None	Not Serious	Not Serious	Not Serious	Not Serious	⊕⊕◯◯ Low
(Siam, 2014)	None	Not Serious	Serious<br>*Not representative of the population (only women from Infertility Clinic)	Very Serious<br>*Confidence Interval and effect size was not calculated and recorded<br>*No adjustment to confounding	Not Serious	⊕◯◯◯ Very Low
(Al-Jefout Et Al., 2017)	None	Not Serious	Not Serious	Not Serious	Not Serious	⊕⊕◯◯ Low
(Al-Jefout Et Al., 2018)	None	Not Serious	Not Serious	Not Serious	Not Serious	⊕⊕◯◯ Low
(Aliani Et Al. 2018)	None	Not Serious	Not Serious	Serious<br>*No adjustment to confounding	Not Serious	⊕◯◯◯ Very Low
(Ashrafi Et Al., 2016)	None	Not Serious	Not Serious	Not Serious	Not Serious	⊕⊕◯◯ Low
(Eggert Et Al., 2008)	None	Not Serious	Not Serious	Not Serious	Not Serious	⊕⊕◯◯ Low
(Eisenberg Et Al., 2018)	None	Not Serious	Not Serious	Not Serious	Not Serious	⊕⊕◯◯ Low
(Lofti Et Al., 2016)	None	Not Serious	Serious<br>*Prevalence estimate was mentioned but it does not state the total sample size	Serious<br>*No control group<br>*No adjustment to confounding<br>*Confidence Interval and effect size was not calculated and recorded	Not Serious	⊕◯◯◯ Very Low
(Mousa, 2019)	None	Not Serious	Serious<br>*Prevalence estimate was mentioned but it does not state the total sample size	Serious<br>*Mean age not reported<br>*No adjustment to confounding<br>*Confidence Interval and effect size was not calculated and recorded	Not Serious	⊕◯◯◯ Very Low

(M. Darwish Et Al., 2006)	None	Not Serious	Not Serious	Very Serious<br>*No adjustment to confounding<br>*Control group not representative of the population	Not Serious	⊕◯◯◯ Very Low
(Obermeyer Et Al., 1986)	None	Not Serious	Serious<br>*Not representative of the population (only women from Infertility Clinic)	Serious<br>*No adjustment to confounding	Not Serious	⊕◯◯◯ Very Low
(Ragab Et Al., 2015)	None	Not Serious	Not Serious	Not Serious	Not Serious	⊕⊕◯◯ Low
(Rouzi Et Al., 2015)	None	Not Serious	Not Serious	Not Serious	Not Serious	⊕⊕◯◯ Low
(Tehrani, Rashidi, et al., 2011)	None	Not Serious	Not Serious	Not Serious	Not Serious	⊕⊕◯◯ Low
(Tehrani, Simbar, et al., 2011)	None	Not Serious	Not Serious	Not Serious	Not Serious	⊕⊕◯◯ Low
(Varghese Et Al., 2012)	None	Not Serious	Not Serious	Not Serious	Not Serious	⊕⊕◯◯ Low
(Yildiz et al., 2012)	None	Not Serious	Not Serious	Not Serious	Not Serious	⊕⊕◯◯ Low
(Abbas Et Al., 2016)	None	Not Serious	Not Serious	Not Serious	Not Serious	⊕⊕◯◯ Low
(Kazemi Et Al., 2014)	None	Not Serious	Not Serious	Not Serious	Not Serious	⊕⊕◯◯ Low
(Maghsudi Et Al., 2012)	None	Not Serious	Not Serious	Serious<br>*Confidence Interval and effect size was not calculated and recorded<br>*No adjustment to confounding	Not Serious	⊕◯◯◯ Very Low
(Al-Kadri Et Al., 2002)	None	Not Serious	Not Serious	Serious<br>*Confidence Interval and effect size was not calculated and recorded<br>*No adjustment to confounding	Not Serious	⊕◯◯◯ Very Low

(Sait Et Al., 2008)	None	Not Serious	Not Serious	Serious<br>*Confidence Interval and effect size was not calculated and recorded<br>*No adjustment to confounding	Not Serious	⊕◯◯◯ Very Low
(Guzel Et Al., 2014)	None	Not Serious	Not Serious	Serious<br>*Confidence Interval and effect size was not calculated and recorded<br>*No adjustment to confounding	Not Serious	⊕◯◯◯ Very Low
(Pity Et Al., 2011)	None	Not Serious	Not Serious	Serious<br>*Confidence Interval and effect size was not calculated and recorded<br>*No adjustment to confounding	Not Serious	⊕◯◯◯ Very Low
(Saleh Et Al., 2013)	None	Not Serious	Not Serious	Serious<br>*Confidence Interval and effect size was not calculated and recorded<br>*No adjustment to confounding	Not Serious	⊕◯◯◯ Very Low
(Yenial Et Al., 2007)	None	Not Serious	Not Serious	Serious<br>*No adjustment to confounding	Not Serious	⊕◯◯◯ Very Low
(Zaid Et Al., 2017)	None	Not Serious	Not Serious	Serious<br>*No adjustment to confounding	Not Serious	⊕◯◯◯ Very Low
